# Tissue- and time-dependent transcription in *Ixodes ricinus* salivary glands and midguts when blood feeding on the vertebrate host

**DOI:** 10.1038/srep09103

**Published:** 2015-03-13

**Authors:** Michalis Kotsyfakis, Alexandra Schwarz, Jan Erhart, José M. C. Ribeiro

**Affiliations:** 1 Institute of Parasitology, Biology Center of the Czech Academy of Sciences, Branisovska 31, 37005 Budweis, Czech Republic; 2 Section of Vector Biology, Laboratory of Malaria and Vector Research, National Institute of Allergy and Infectious Diseases, 12735 Twinbrook Parkway room 2E32D, Rockville MD 20852, USA

## Abstract

*Ixodes ricinus* is a tick that transmits the pathogens of Lyme and several arboviral diseases. Pathogens invade the tick midgut, disseminate through the hemolymph, and are transmitted to the vertebrate host via the salivary glands; subverting these processes could be used to interrupt pathogen transfer. Here, we use massive *de novo* sequencing to characterize the transcriptional dynamics of the salivary and midgut tissues of nymphal and adult *I. ricinus* at various time points after attachment on the vertebrate host. Members of a number of gene families show stage- and time-specific expression. We hypothesize that gene expression switching may be under epigenetic control and, in support of this, identify 34 candidate proteins that modify histones. *I. ricinus*-secreted proteins are encoded by genes that have a non-synonymous to synonymous mutation rate even greater than immune-related genes. Midgut transcriptome (mialome) analysis reveals several enzymes associated with protein, carbohydrate, and lipid digestion, transporters and channels that might be associated with nutrient uptake, and immune-related transcripts including antimicrobial peptides. This publicly available dataset supports the identification of protein and gene targets for biochemical and physiological studies that exploit the transmission lifecycle of this disease vector for preventative and therapeutic purposes.

I*xodes ricinus* is a European tick vector responsible for transmitting the microbial agents that cause Lyme disease and several arboviral diseases including the serious tick-borne encephalitis^1^,^2^. The life cycle of these pathogens within the tick involves invasion of midgut cells, dissemination through the hemolymph, and invasion of the salivary glands, from where they are delivered to their vertebrate hosts within salivary secretions. Tick saliva serves multiple functions during blood feeding including acting as a cement to adhere the tick to the host skin and counteracting the hemostatic, inflammatory, and immune reactions of the host that could disrupt feeding^3^.

Next generation deep transcriptome sequencing (and the subsequent assembly of the sequence reads into transcript contigs) allows the simultaneous identification of many thousands of coding sequences, while comparisons between the different tissue libraries sequenced allow the identification of tissue-specific transcription. Libraries prepared from tissues at different physiological time points can also help to identify the succession of transcripts, for example as the tick midgut or salivary glands change from a resting state to one of full activity. We have previously reported the results of a low-throughput gene discovery project based on normalized salivary gland libraries of *I. ricinus* to characterize the entire life cycle of this tick^4^, and in doing so vastly expanded the known repertoire of salivary gland protein sequences in *I. ricinus*^5^. We also created an *I. ricinus* salivary transcript database to support the simultaneous study of the proteomic and transcriptomic dynamics of almost 1,500 genes and their products^6^ which revealed that proteomic and transcriptomic dynamics do not agree in the case of some genes.

Subverting the normal tick feeding process could be exploited to disrupt pathogen transfer between the vector and vertebrate host. The importance of revealing the tissue specificity in gene expression upon an infectious meal of the tick *I. ricinus* with the pathogen *Bartonella henselae* and of the tick *Rhipicephalus (Boophilus) microplus* with the parasite *Babesia bovis* has already been demonstrated^7^,^8^,^9^. Here, we aim to describe the transcriptional changes occurring in the salivary and midgut tissues of nymphal and adult *I. ricinus* following attachment and blood feeding to the vertebrate host. In doing so, we increase the number of known transcripts in this tick and develop insights into gene regulatory mechanisms. The pattern of early salivary gland transcription reveals possible immunogens that could be used in vaccine development for early tick removal prior to pathogen delivery. The identification of salivary gland transcripts aids the future discovery of biologically active components that account for the properties of tick saliva, while the identification of midgut transcripts furthers our understanding of the receptors and proteins that regulate pathogen uptake, innate immunity, and digestion.

## Results and Discussion

### Overall characteristics of the salivary and midgut transcriptomes of *Ixodes ricinus*

**Figure 1:**
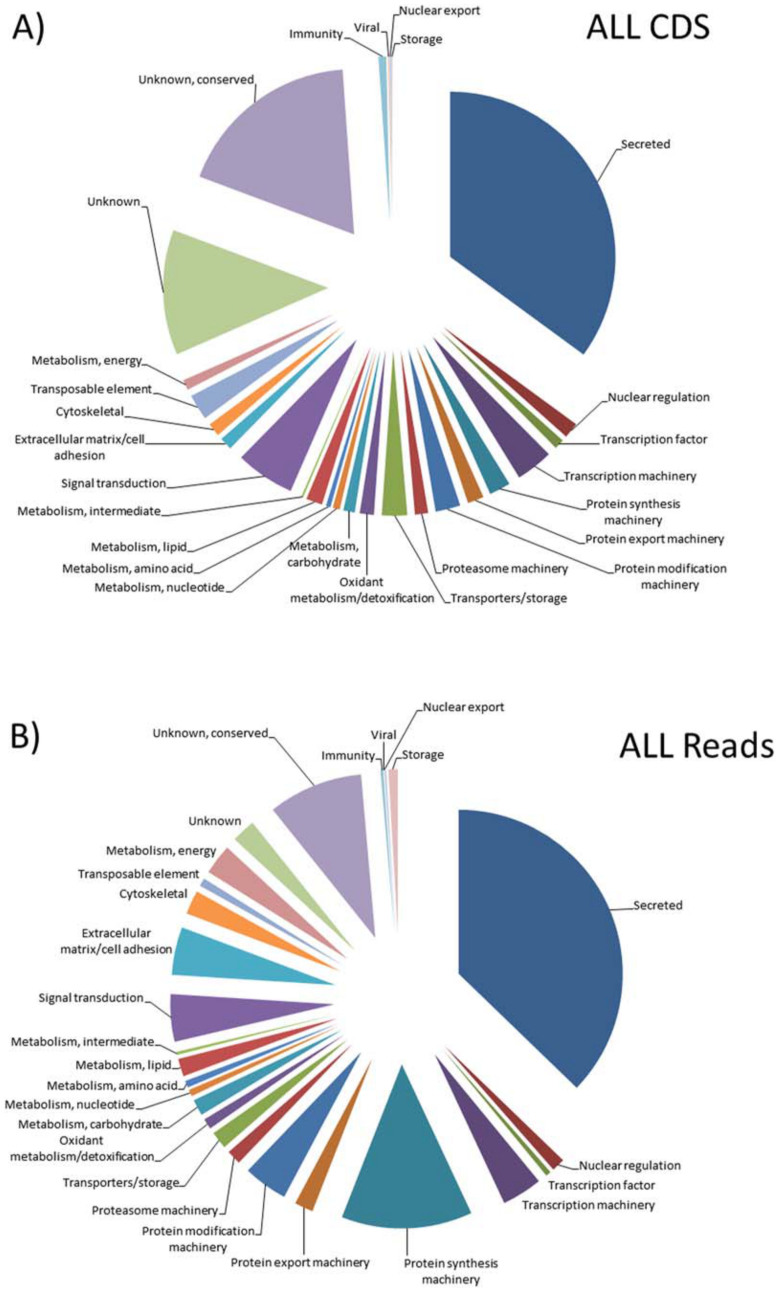
The genes belonging to different functional classes in the salivary gland and midgut transcriptomes of *Ixodes ricinus*. Depending on the predicted function of the polypeptides for which the coding sequences (CDS) encode, two different pie charts are shown. In (A), the proportion of the total number of different CDS encoding a polypeptide of the same predicted function are compared to the total number of CDS found in our transcriptomes. In (B), the proportion of the total number of different sequence reads assigned to CDS encoding a polypeptide of the same predicted function are compared to the total number of mapped sequence reads in our transcriptome.

Ten non-normalized Illumina libraries were prepared from total RNA isolated from *I. ricinus* salivary glands or midguts dissected from female adult ticks (fed on the vertebrate host for either 12, 24, or 36 h) or nymphal ticks (fed on the vertebrate host for either 12 or 24 h). Sequencing of the ten different libraries (see Materials and Methods for details) produced 268,914,130 Illumina sequence reads that were *de novo* assembled into contigs along with 441,381,454 pyrosequencing reads and 67,703,183 Illumina reads from our previous work^4^. Extraction of the coding sequences (CDS) from the raw data assembly generated 25,808 CDS larger than 150 nucleotides (nt), to which the reads of the ten libraries from nymphal and adult salivary glands and midguts were mapped to identify tissue- and time point-specific transcriptional patterns (Supplemental File S1). A total of 148,813,058 reads (55% of total library reads) were thus mapped on the 25,808 CDS. The CDS were classified into five general categories following automatic annotation and manual curation, namely secreted (S), housekeeping (H), transposable elements (TE), viral (V), and unknown (U) (Supplemental Table 1). The S class contained almost one third of the total contigs and reads, while the H class contained half of the total CDS and 60% of the total reads; together, these classes accounted for 85% of the CDS and over 96% of the mapped reads. The S class was further subdivided into 54 functional classes (see Supplemental Table 2) as previously described^4^,^10^,^11^, with basic tail, Kunitz, and lipocalin proteins accounting for 6.4% of the 25,508 CDS and 12.1% of the total reads. In the H class (Supplemental Table 2), the protein synthesis machinery class accounted for the majority of reads, followed by unknown conserved, extracellular matrix, signal transduction, and protein modification machinery. Functional classification of the transcriptome by CDS number (Figure 1A) or number of reads (Figure 1B) highlights the complexity and depth of the resulting assembly and helps to visualize the significant metabolic investment occurring in these tissues during feeding. For example, the protein synthesis machinery class has a relatively small number of coding sequences represented, but comparatively greater “mass” as represented by the number of reads. Conversely, the unknown category has a larger number of putative CDS but a relatively small number of accumulated reads (Figure 1). The hyperlinked spreadsheets with transcript annotations and dynamics (Supplemental Files S1, S2, and S3) are available from http://exon.niaid.nih.gov/transcriptome/Ixric-sgmg/Ixric-sgmg-S1-web.xlsx, http://exon.niaid.nih.gov/transcriptome/Ixric-sgmg/Ixric-sgmg-S2-web.xlsx, and http://exon.niaid.nih.gov/transcriptome/Ixric-sgmg/Ixric-sgmg-midgut-specific-S3-web.xlsx. All the sequences can be seen by double clicking on the name of each transcript in column A of the spreadsheets.

**Figure 2:**
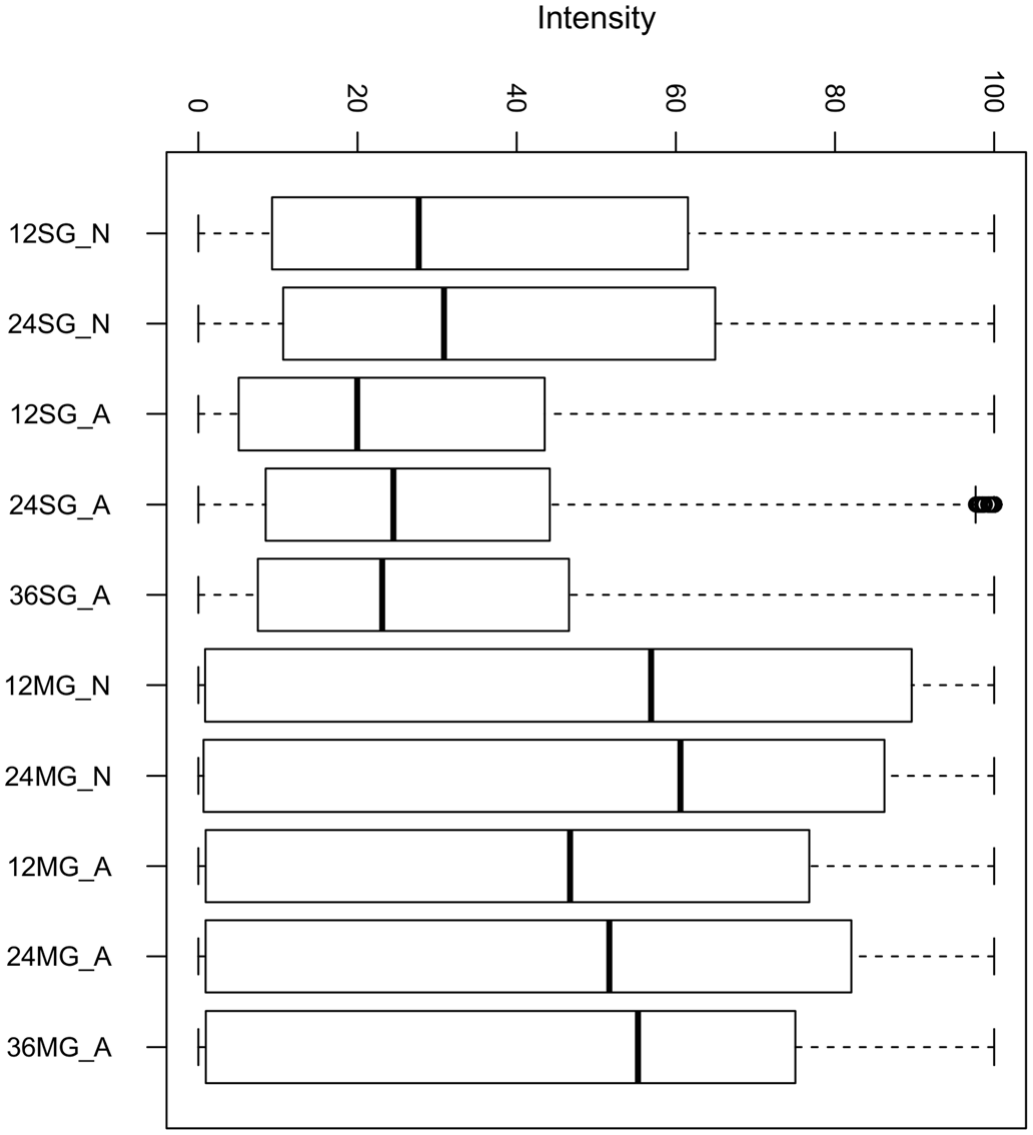
The breadth of tissue- and time-dependent differential expression. A box plot was constructed using normalized reads per kilobase per million (RPKM) values for CDS with a total RPKM (considering the reads of all ten libraries) of 50 or larger to avoid inclusion of poorly expressed contigs. The normalized RPKM values (maximum 100) for ten different libraries from *Ixodes ricinus* are as follows: the first two numbers indicate the time point of organ collection from 0–12 h (12), 12–24 h (24), or 24–36 h (36). Organs were either salivary glands (SG) or midguts (MG) and developmental stage was either nymphs (N) or adults (A). For more details, please consult the methods.

**Figure 3:**
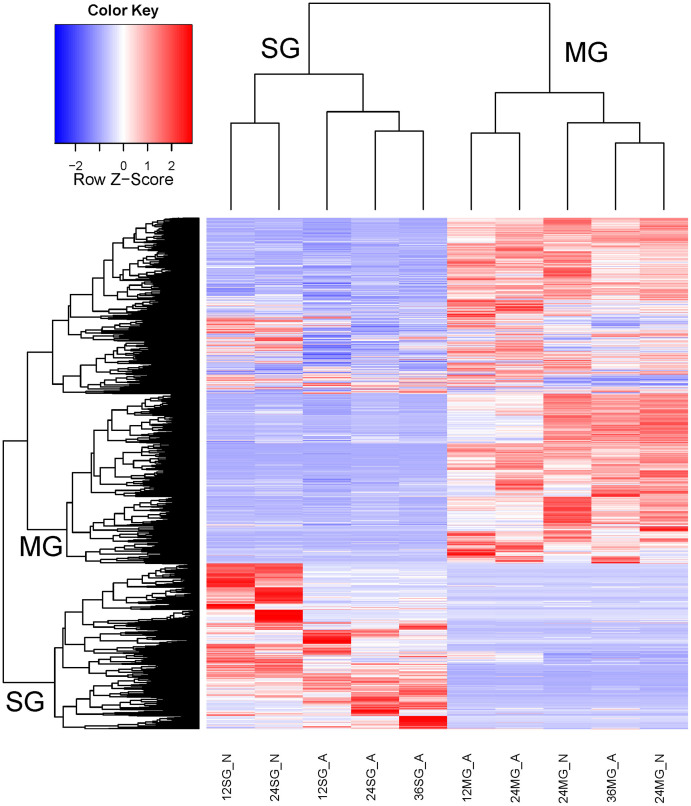
Heat map of normalized RPKM data from the salivary glands (SG) and midguts (MG) of nymphal and adult *Ixodes ricinus* fed for different periods of time. The Z score represents the deviation from the mean by standard deviation units. For other details, please see the Figure 2 legend.

To visualize the full breadth of differential expression between tissues and time points, a box plot and heat map were constructed using normalized reads per kilobase per million (RPKM) values for CDS with a total RPKM of 50 or greater (considering the reads of all ten libraries) to avoid inclusion of poorly expressed contigs. For this set of 11,775 CDS, RPKM values for each library were divided by the largest value and multiplied by 100. The salivary gland (SG) reads had a smaller average and larger dispersion than the midgut (MG) reads (Figure 2). The corresponding heat map (Figure 3) reveals clear clustering of SG or MG CDS according to expression. The lower left corner of the heat map shows CDS highly expressed in the SG at particular times points post feeding or developmental stage, while the upper right corner indicates midgut transcripts that are transcribed in different tissues or at different time points.

**Figure 4:**
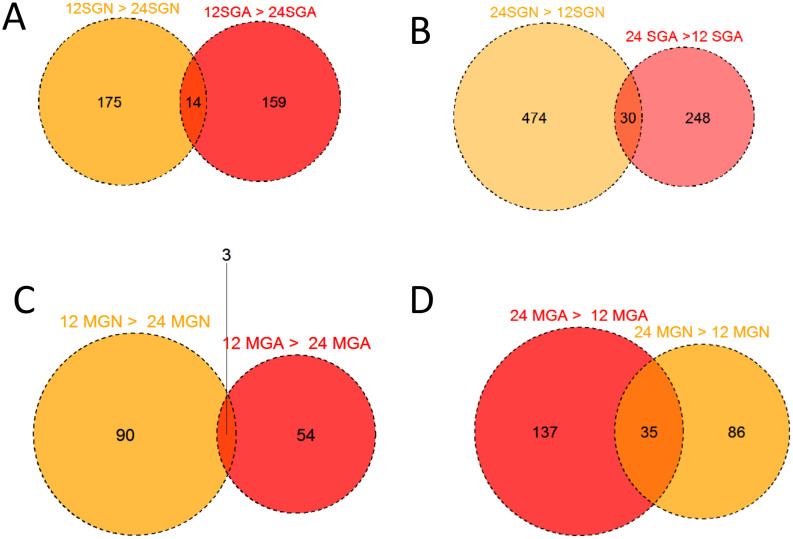
Venn diagrams representing pairwise comparisons of transcripts at least ten-times (10×) overexpressed in the salivary glands (SG) or midguts (MG) of nymphal (N) or adult (A) *Ixodes ricinus* ticks fed for up to 12, or from 12 to 24 hours, on a host. (A) Comparison of transcripts 10× overexpressed at 12 h vs. 24 h of feeding in nymphs and adults. (B) Same as A, but transcripts overexpressed at 24 h vs. 12 h. (C) Same as A, but using MG instead of SG. (D) Same as B, but using MG instead of SG.

Tissue- and time-specific transcription was also evident by comparing the number of mapped reads from library pairs. Thus, 5,406 CDS were ten times or more overexpressed in the combined SG than MG libraries and 2,943 CDS in the reverse comparison, indicating putative SG- or MG-specific transcripts (Table 1) and corroborating the heat map results (Figure 3). Of the 5,406 transcripts overexpressed in SGs, 4,332 (80%) belonged to the S class (see Supplemental Table S3 for details), compared to only 733 (24%) S class overexpression in the MG (Supplemental Table S4). Other pairwise comparisons are also presented in Table 1. With respect to early expressed SG transcripts, 189 CDS were overexpressed at 12 h vs. 24 h in nymphs, with similar numbers (173) overexpressed at 12 h in adults; however, only 14 were common to both groups (Figures 4A and B) indicating that gene profiles in adults and nymphs are different at early time points. Similarly, a comparison of transcripts overexpressed at 24 h compared to 12 h also revealed a small number of common CDS between nymphs and adults, again indicating specialization of gene expression at each developmental stage. Despite the secreted class representing less than 50% of the total CDS and number of reads, they represented the majority of overexpressed CDS in all paired SG time-dependent comparisons in both nymphs and adults (Supplemental Figure S1). A similar pattern was found for time-dependent MG expressions (Figures 4C and D and Supplemental Figure S2). Furthermore, while hundreds of CDS were overexpressed in all SG library comparisons, the adult MG became more stable at 36 h when only a few CDS appeared uniquely expressed compared to the adult MG at 24 h (Supplemental Figure S3). On the other hand, over 300 CDS were at least ten-times overexpressed at 36 h compared to the 24 h library in the adult SG. The SG appears to be a lot more transcriptionally labile with continuous appearance of new transcripts, particularly of the secreted class, while the adult midgut stabilizes its gene expression at this time point, indicating it reached a functional steady state.

### Functional classification and expression pattern of salivary transcripts associated with time-dependent expression

**Figure 5:**
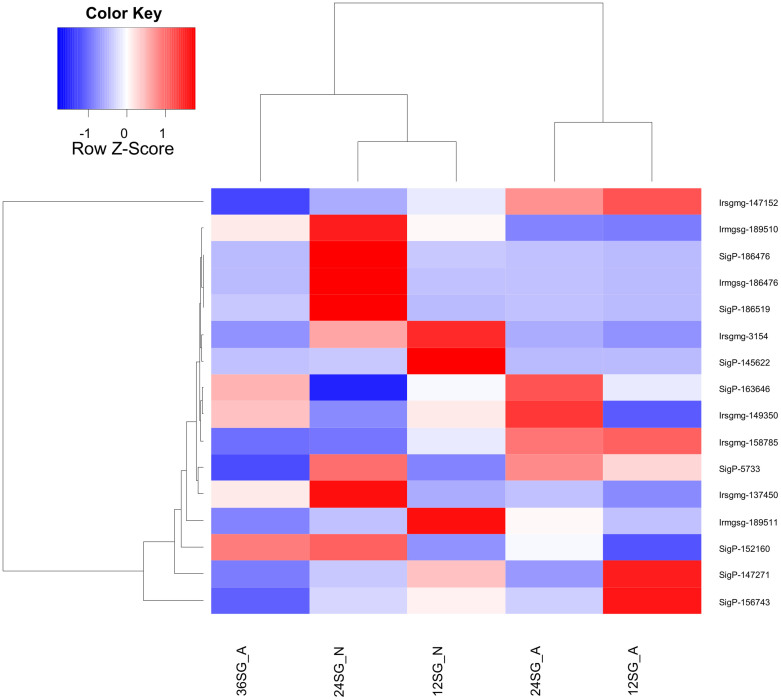
Genes encoding salivary metalloproteases in *Ixodes ricinus* that are at least ten-times differentially expressed at different tick developmental stages (nymphs or adults) and times post feeding (12, 24, or 36 hours).

A total of 1,447 salivary CDS were at least ten-times overexpressed in all time-dependent pairwise comparisons (Supplemental Figure S3). Their detailed functional classification is shown in Supplemental Table S5. Of these, 1,135 (77%) belonged to the S class and included 212 CDS for Kunitz domain-containing peptides, 97 members of the Salp 15/ixostatin superfamily, 95 lipocalins, 16 metalloproteases, and several other recognizable families of unknown function. Kunitz, lipocalin, and metalloproteases tended to be uniquely expressed at a particular developmental stage (nymph or adult) and at particular times (Figure 5 and Supplemental Figures S4 and S5).

### Time-dependent functional classification and expression patterns of midgut transcripts

A total of 440 midgut CDS were at least ten-times overexpressed in all time-dependent pairwise comparisons (Supplemental Figure S3). 269 (61%) of these were classified as S class, including 38 Kunitz domain-containing peptides, 28 basic tail peptides, and 11 lipocalin family peptides; these peptides are typically found in tick sialotranscriptomes (Supplemental Table S6)^10^. 83 of the 440 overexpressed CDS were the same in both the SG and MG (Supplemental Figure S6). Of these, 74 (89%) belonged to the S class, including 15 Kunitz members and two lipocalins. These large protein families typically found in sialotranscriptomes also appear to be differentially expressed in the tick midgut. Indeed, a five Kunitz domain-containing protein showing low salivary expression has been identified in tick midguts, which is important for *Rickettsia* virulence^12^,^13^.

### Insight into the midgut transcriptome (mialome) of *Ixodes ricinus*

Although the sialome of ticks is reasonably well described, including in *I. ricinus*, few studies have focused on midgut transcriptome, also known as the ‘mialome'^14^. Here, we highlight CDS that appear to be related to midgut function, namely the 2,943 CDS expressed ten-times or more in the MG compared to the SG (Supplemental File S3).

#### Enzymes

**Figure 6:**
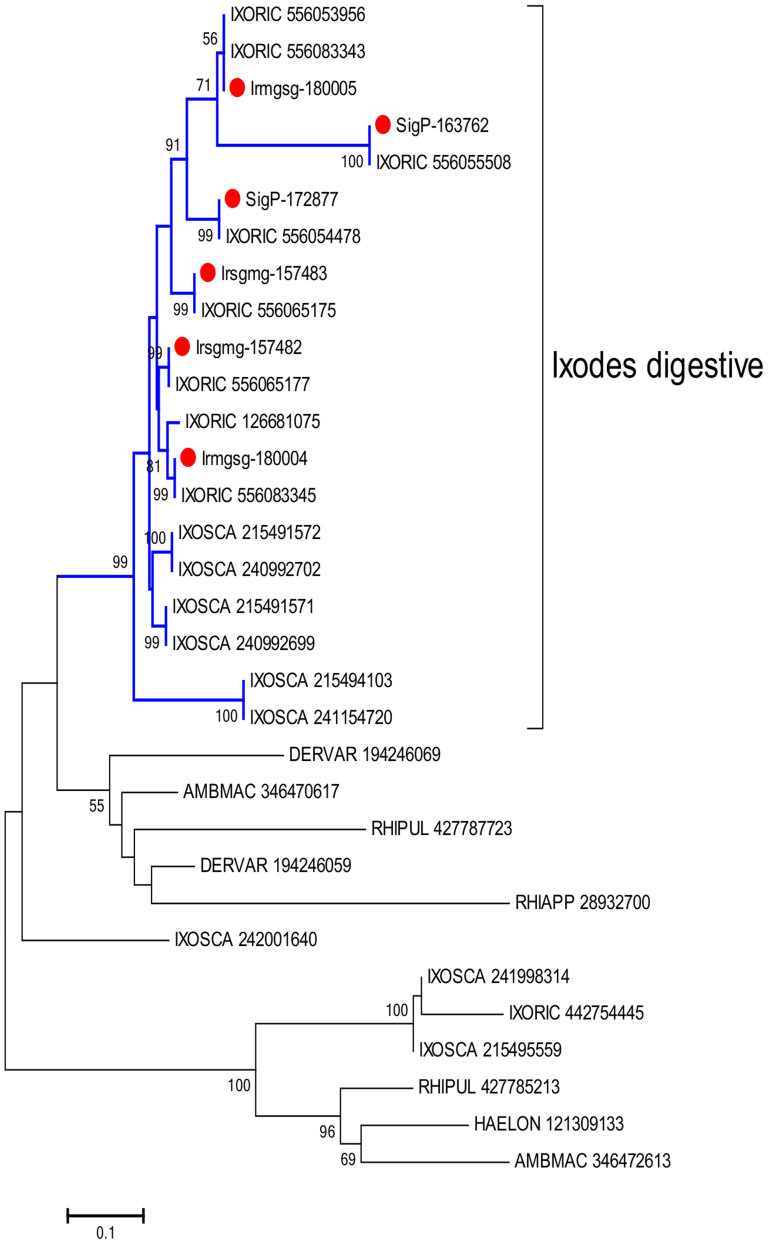
Phylogeny of the cathepsin B family of tick proteases. Multiple protein sequence alignments of all the cathepsin B proteins from ticks was used to construct a phylogenetic tree of the specific family. The red symbols indicate the six enzymes overexpressed in the midgut of *Ixodes ricinus* (found as overexpressed in this work) beside their GenBank deposited sequence references. GenBank sequences are recognizable by the three first letters of their genus name followed by the first three letters of the species name followed by their GenBank accession number (IXORIC: Ixodes ricinus, IXOSCA: Ixodes scapularis, DERVAR: Dermacentor variabilis, AMBMAC: Amblyomma maculatum, RHIPUL: Rhipicephalus pulchellus, RHIAPP: Rhipicephalus appendiculatus, HAELON: Haemaphysalis longicornis). The numbers near the branches indicate the percentage of bootstrap support using the neighbor joining algorithm. Values lower than 50 are not shown. The bar at the bottom indicates 10% amino acid substitution rate.

In ticks, meal digestion is intracellular and requires endocytosis and the action of lysosomal enzymes^15^. Proteins are digested via a multi-enzyme pathway that has been characterized by proteomic analyses using peptidase inhibitors^16^. These studies have identified cathepsins B, C, and D, legumain, and serine carboxypeptidase and leucine aminopeptidase as the main enzymes acting in the hemoglobinolytic system of *I. ricinus*. The MG transcriptome libraries were enriched for six cathepsin B enzyme-coding transcripts, which were present in all the MG libraries and had high levels of expression (RPKMs between 1,300 and 19,000). Their phylogeny is represented in Figure 6. A single well-expressed CDS coding for a cathepsin D (RPKM 2453) was also identified, 96% identical to the *I. scapularis* homolog. Four papain-like cathepsin L CDS were identified, coding for enzymes of ~335 amino acids. Two of these were overexpressed in MG vs. SG libraries, with only 72% identity suggesting derivation from two genes. Another CDS coded for a 558 amino acid pro-enzyme with 63% identity to a midgut cysteine proteinase from *R. appendiculatus* (gi|28932702)^17^. Three full-length legumains (RPKMs between 250 and 6,500) were also identified; these had only 60% identity, again indicating multiple gene origin. Thirty CDS coding for serine proteases were overexpressed in the MG libraries, 21 of which were over 200 amino acids in length. Of these, six coded for large proteins (>400 amino acids) and included a CUB domain. Three were highly expressed, including SigP-158745 (RPKM 12,500; 387,923 reads), which had 50% identity to a cubilin serine protease in *Haemaphysalis longicornis* (gi|47847115)^18^. These *I. ricinus* cubilins appear to be derived from multiple genes, since they shared only 44% identity. Six additional serine proteases were highly expressed in the tick MG (RPKMs 600 to 4,300) and also appeared to be derived from multiple genes, since they had less than 70% identity. The MG was also enriched for large metalloproteases, including an astacin-like metalloprotease with an RPKM of 3,013. A full-length dipeptidyl peptidase (cathepsin C) was identified (RPKM 2,963). Several carboxypeptidases were also found; for example, SigP-149623 codes for a zinc carboxypeptidase with an RPKM of 9,305 (from 94,159 sequence reads). Several serine carboxypeptidases were identified, six of which were full length or near full length (based on multiple sequence alignments, data not shown). Their alignment and dendrogram indicate that they are derived from multiple genes (data not shown).

The exact mechanism of lipid digestion in ticks remains elusive, although ultrastructural work has shown lipid accumulation in MG cells as blood digestion progresses^19^,^20^. Forty-four CDS coding for lipases were overexpressed in *I. ricinus* MG libraries, 15 of which with an RPKM greater than 500 and 12 greater than 1,000. These included several pancreatic lipase-like enzymes, phospholipase A2, and sphingomyelinases. Similarly, the enzymes involved in tick carbohydrate digestion are unknown; several putative carbohydrate-metabolizing enzymes with elevated RPKMs are shown in Supplemental File S3 and include alpha-amylase, beta-N-acetylhexosaminidase, beta-galactosidases, alpha-fucosidase, alpha-glucosidase, and alpha-galactosidase. A chitinase and chitolectin-chitotriosidase were also found in the transcriptomes, which may be involved with peritrophic matrix modeling. With respect to DNA digestion, a CDS coding for a full-length DNAse II was highly MG specific (MG RPKM of 4,413 vs. SG RPKM of 10).

#### Immunity-related products

Transcripts coding for antimicrobial peptides of the defensin, microplusin, and lysozyme families were overexpressed in the MG libraries, along with transcripts coding for peptidoglycan recognition proteins and ML-domain containing peptides; these may function as pathogen recognition receptors or as lipid carriers in lysosomes. Transcripts coding for cytokines and cytokine receptors were also identified.

#### Protease inhibitors

Peptides with TIL, Kunitz, and cystatin domains and serpins were highly expressed in MGs, including many with high RPKMs and over 1,000-fold overexpression in relation to the SG. As mentioned above, this suggests that some families classically thought to be SG-restricted, as previously reviewed in Ref. 10 are also MG abundant, with some members being MG specific, at least at the transcriptional level.

#### Cytotoxin and 13 kDa family

Several MG-specific transcripts coding for proteins with an ETX_MTX2 PFAM domain derived from *Clostridium epsilon* mosquitocidal toxin MTX2 were identified with high RPKMs and several hundred-fold overexpression compared to the SG. Although their function is unknown, they may be associated with the hemolytic activity previously described in *I. scapularis* midguts^21^. Similarly, many transcripts coding for members of the 13 kDa family of salivary proteins previously found in *Ixodes* salivary glands were overexpressed in the MG.

#### Other secreted proteins

An additional 583 transcripts coding for putative secreted proteins and overexpressed in the MG are shown in Supplemental File S3. Many of these were short in size and may therefore represent artifactual CDS derived from non-coding RNAs.

#### Chitin deacetylase and chitin-binding domains

One predicted peptide was found to contain the PFAM CBM_14 chitin-binding peritrophins-A domain, two coded for chitin synthases, and 42 had the CDD CE4_CDA_like_2 domain (the putative catalytic domain of chitin deacetylase-like proteins). These proteins may be involved in peritrophic matrix formation and maintenance.

#### Detoxification

Over 90 transcripts overexpressed in the MG coded for members of the cytochrome P450 family, at least 30 of which appeared to be full length with lengths over 400 amino acids. Glutathione transferases, selenoproteins, and sulfotransferases were also found.

#### Signal transduction

This functional class was highly represented in MG-overexpressed transcripts, with 325 members ten-times or more overexpressed in the MG vs. SG libraries. Twelve code for 7 transmembrane domain G-coupled receptors that might mediate agonist hormone activity on MG cells or their musculature.

#### Storage

Ferritin, vitellogenins, and vitellogenin receptors were overexpressed in MG libraries, including SigP-192477, which codes for a full-length ferritin and was assembled from 118,002 reads, nearly all originating from the MG libraries. Some vitellogenins were well expressed in both adults and nymphs and could be linked to lipid transport rather than acting as an egg yolk protein.

#### Transcription factors

The depth of sequencing allowed for full-length retrieval of several transcription factors overexpressed in the MG, including GATA, MYB, E2F7, forkhead, and several others.

#### Transporters

As expected, a large number (132) of transporters and channels were overexpressed in MG tissues. The most overexpressed included an organic cation/carnithine transporter (MG RPKM = 767, SG RPKM = 3.4), an ABC transporter (MG RPKM = 725, SG RPKM = 1.1), and an epithelial chloride channel (MG RPKM = 577, SG RPKM = 0.7). RNAi experiments against some of these proteins could identify targets for digestion disruption in *I. ricinus*.

#### Transposable elements

Two NLTR retrotransposons were highly expressed in the MG (MG RPKMs 906 and 1021, SG RPKM < 5), which may represent domesticated retroposons associated with gene expression control^22^. Sixty-five other transposons were overexpressed in the MG and mostly represented fragments with small RPKMs (5–50).

#### Viral sequence

A predicted 611 amino acid protein had 35% identity and 57% similarity to the *Aguacate virus* glycoprotein. This protein also matches with the PFAM domain Phlebovirus_G2 with an E value of 1e-127. This contig was assembled from 50 reads from the MG libraries and none from the SG and had a small RPKM = 0.86. Whether this viral sequence is inserted in the tick genome remains to be elucidated.

### Possible gene switching mechanisms underlying differential gene expression within gene families

It is possible that epigenetic mechanisms, such as histone methylation or acetylation, regulate the time- or stage-dependent transcriptional switching of genes as in embryonal development^23^,^24^ or switching of endothelial adhesive proteins in *Plasmodium*^25^. The tick genes associated with salivary gland function may have increased over time via episodes of genome duplication in addition to the more common mechanism of tandem repeat duplications, allowing different genomic regions to contain subsets of functionally similar but divergent genes with different antigenic properties. Indeed, tick genomes are large: *I. scapularis*'s genome is larger than that of the chicken^26^, and metastriate ticks have larger genomes than humans^27^. Histone modifications at different genomic regions may, therefore, regulate gene transcription. Here, 34 transcripts coding for proteins associated with histone modifications were identified, including two near full-length histone deacetylase components (RPD3) and six sirtuins (NAD-dependent protein deacetylases). Seven transcripts coded for proteins with SET domains characteristic of histone lysine methyltransferases, two of which were ten-times or more overexpressed in the MG. Although some of these CDS were not full length, their sequences could still be used to design RNAi experiments to investigate the functional role of these genes in the control of gene transcription in *I. ricinus*. The CDS and protein sequences are available on Supplemental File S1 (worksheet named ‘Epigenetics').

### Polymorphisms analysis

Previous work in mosquitoes has indicated that genes encoding salivary proteins in hematophagous insects have higher numbers of polymorphisms and non-synonymous substitutions than other gene classes^28^,^29^. Mapping the reads on the assembled contigs allowed us to determinate single nucleotide polymorphisms (SNPs) in the CDS. For this analysis, a subset of 13,910 CDS with an average read depth of 50 or more was used to avoid inclusion of low read-depth assembled sequences. Immunity, secreted, and unknown classes had the highest rate of non-synonymous substitutions per codon and the highest non-synonymous/synonymous ratios, suggesting accelerated evolution of these gene classes (Table 2).

## Conclusions

In this paper, we *de novo* assembled 441,381,454 pyrosequencing reads and 67,703,183 Illumina reads obtained from normalized libraries of nymphal and adult *I. ricinus* from our previous work^4^ together with 268,914,130 reads from ten non-normalized libraries from nymphal and adult salivary glands and midguts. 25,808 CDS larger than 150 nt were extracted in total. Mapping only the non-normalized reads coming from this work (the reads from Ref. 4 were not mapped) to the deduced CDS allowed detailed analysis of tissue- and time-specific gene expression. Tick salivary proteins contain many protein families including the lipocalins, Kunitz-domain containing proteins, and metalloproteases, and members of these families show stage and time-specific expression (Figure 5 and Supplemental Figures S4 and S5). Members of these families can be very diverse, and it has been suggested that such selective expression may represent a mechanism of antigenic variation^30^. We hypothesize that gene expression switching may be under epigenetic control, and the identification of 34 candidate proteins involved in histone modification supports this notion. Secreted proteins are coded for by genes with a high rate of non-synonymous to synonymous mutations, even greater than immune-related genes, indicating fast evolution of this class.

We analyzed the mialome of *I. ricinus* in detail, and in doing so describe several enzymes associated with protein, carbohydrate, and lipid digestion, along with transporters and channels that might be related to nutrient intake. This work is limited by the focus on only two tick tissues and the lack of biological replication due to cost constraints, and future work will first require validation of these findings. Nevertheless, the reported results are biologically intuitive: Transcripts known to be associated with salivary gland function were expressed at hundred-fold higher levels than midgut transcripts, and known digestive enzymes were expressed at orders of magnitude higher in the midgut compared to the salivary glands. This study represents a valuable mining platform for future studies involving the salivary glands and midgut biochemistry and physiology of *I. ricinus* and helps broaden our understanding of the molecular events mediating the transmission lifecycle of this important disease vector.

## Methods

### Ethics statement

All animal experiments were carried out in accordance with the Animal Protection Law of the Czech Republic (§17, Act No. 246/1992 Sb) and with the approval of the Akademie Ved Ceské Republiky (approval no. 161/2010).

### Ticks, tissue dissection, total RNA/protein isolation, and sequencing

Briefly, 1,080 nymphs and 420 adult females and males were attached to experimental animals for feeding. mRNA was extracted from pools of tissues dissected from female adult ticks and nymphal ticks feeding for 3 hour periods up to 24 hours (nymphs) or 36 hours (adults), providing ten samples as follows: four samples for 0–12 h and 12–24 h for nymphal salivary glands (SG) and midguts (MG), and six samples for 0–12 h, 12–24 h, and 24–36 h for adult SG and MG. These non-normalized libraries were sequenced using an Illumina HiSeq 2000 machine and the raw data deposited at the Sequence Read Archives (SRA) of the National Center for Biotechnology Information (NCBI) under accessions SRR641305, SRR641306, SRR641307, SRR641308, SRR641309, SRR641327, SRR641328, SRR641329, SRR641330, and SRR641331 (Supplemental Table S1).

### Read assembly and bioinformatics

The reads indicated above, plus reads derived from our previous work (SRA accessions SRR592662, SRR592663, SRR592664, SRR592665, SRR592674, SRR592675, SRR592676, and SRR592677)^4^, were reassembled using Abyss^31^,^32^ and SOAPdenovo-Trans^33^ with various k values, and the assembly of the assemblies was performed with 15 iterations of a blastn and cap3 pipeline^34^ to generate 198,504 contigs larger than 150 nt with an average size of 662 nt and L50 = 501 (Supplemental Table S2). Coding sequences (CDS) were extracted based on larger open reading frames (ORFs) containing a signal peptide and by translated contigs that matched protein sequences derived from the Swiss-Prot, invertebrate RefSeq, and GenBank-extracted protein sequences with “acari” in their organism name. From these CDS, 16,002 have been deposited via the Transcriptome Shotgun Assembly (TSA) portal of the NCBI to DDBJ/EMBL/GenBank under the accession GANP00000000 and BioProject ID PRJNA217984. The version described in this paper is the first version, GANP01000000. The non-redundant set of CDS was mapped to a hyperlinked Excel spreadsheet that charts these sequences to several database comparisons and the number of reads derived from each of the ten libraries^28^,^34^. RPKM (reads per thousand nucleotides per million reads) values for each sequenced library were calculated for each CDS and included in the spreadsheet as defined by the TopHat/Cufflinks program^35^. The number of reads for each library was compared using the χ^2^ test, and the ratio of reads was calculated by dividing the number of reads derived from one library by the number of reads from the second library (plus one to avoid division by zero) and further multiplying or dividing the result by the ratio of the total reads from each library to normalize the results. For visualization of synonymous and non-synonymous sites within coding sequences, the BWA aln tool^36^ was used to map the reads to the CDS, producing SAI files that were joined with the BWA sampe module, converted to BAM format, and sorted. The sequence alignment/map tools (samtools) package^37^ was used to perform the mpileup of the reads (samtools mpileup), and the binary call format tools (bcftools) program from the same package was used to make the final vcf file containing the single-nucleotide polymorphic (SNP) sites using –D100 and base quality 13 or better (default). Determination of whether the SNPs led to synonymous or non-synonymous codon changes was achieved using a Visual Basic program written by JMCR, the results of which were mapped into the Excel spreadsheet and color visualized in hyperlinked rtf files within Supplemental File S1. Finally, heatmaps were produced with the gplots and heatmap.2 programs in R.

### Availability of supporting data

The raw non-normalized library data were deposited in the Sequence Read Archives (SRA) of the National Center for Biotechnology Information (NCBI) under accessions SRR641305, SRR641306, SRR641307, SRR641308, SRR641309, SRR641327, SRR641328, SRR641329, SRR641330, and SRR641331. Following assembly, a total of 16,002 coding sequences and their protein translations were deposited in DDBJ/EMBL/GenBank under the accession GANP00000000 and BioProject PRJNA217984. The version described in this paper is the first version, GANP01000000. The hyperlinked spreadsheets with transcript annotations and dynamics (Supplemental Files S1, S2, and S3) are available from http://exon.niaid.nih.gov/transcriptome/Ixric-sgmg/Ixric-sgmg-S1-web.xlsx, http://exon.niaid.nih.gov/transcriptome/Ixric-sgmg/Ixric-sgmg-S2-web.xlsx, and http://exon.niaid.nih.gov/transcriptome/Ixric-sgmg/Ixric-sgmg-midgut-specific-S3-web.xlsx.

## Author Contributions

M.K. conceived the study, participated in the design and coordination of the study, and helped to draft the manuscript. A.S. participated in the experimental design, tick dissections, preparation of libraries, and approval of the final manuscript. J.E. participated in the preparation of libraries and approval of the final manuscript. J.M.C.R. performed the bioinformatics analysis and wrote the majority of the manuscript.

## Supplementary Material

Supplementary Information Supplementary information

## Figures and Tables

**Table 1:** Number of coding sequences ten-fold or more overexpressed in the indicated pairwise comparisons according to the number of mapped reads from the indicated libraries

Comparison	Number of sequences
SG > MG	5,406
MG > SG	2,943
12SGN > 24SGN	189
24SGN > 12SGN	504
12SGA > 24SGA	173
24 SGA > 12 SGA	278
24 SGA > 36 SGA	255
36SGA > 24SGA	329
12 MGN > 24 MGN	93
24 MGN > 12 MGN	121
24 MGA > 36 MGA	67
36 MGA > 24 MGA	5

SG = salivary glands, MG = midgut, A = adult, N = nymph, numbers indicate the number of hours post host attachment that the tissues were collected. SG > MG indicates comparison for SG transcripts significantly 10 fold or more expressed in the SG than in MG tissues, and so on.

**Table 2:** Rate of synonymous (Syn) and non-synonymous (NS) nucleotide substitutions per 1,000 codons in different functional classes for 13,910 coding sequences with a read depth greater than 50 per nucleotide site

Class	Average Syn/1000 codons	SE	Average NS/1000 codons	SE	Average NS/Syn+.1	SE	Average coverage depth	SE	N
Proteasome machinery	3.0477	0.2157	0.6539	0.0962	0.2011	0.0403	1,284	276	280
Nuclear regulation	4.0364	0.2516	0.8454	0.0854	0.2038	0.0328	972	227	332
Signal transduction	3.0428	0.1206	0.7344	0.0447	0.2293	0.0202	581	47	1140
Transporters and channels	2.3329	0.1688	0.6717	0.0794	0.2490	0.0416	621	69	417
Cytoskeletal	3.1665	0.2434	0.7611	0.0936	0.2547	0.0486	1,332	219	264
Protein export	3.6622	0.2639	0.7425	0.0850	0.2682	0.0445	1,284	276	342
Transcription Factor	2.9252	0.2712	1.1889	0.1877	0.2931	0.0516	374	70	170
Detoxification	2.3616	0.2578	1.0858	0.1382	0.3107	0.0565	915	121	210
Extracellular matrix	2.1527	0.2173	0.8436	0.1186	0.3387	0.0658	2,596	540	239
Transcription machinery	3.7351	0.1608	1.1853	0.0838	0.3430	0.0321	824	92	751
Metabolism	3.3969	0.1407	1.1396	0.0607	0.3525	0.0305	1,934	199	1016
Protein synthesis	2.5993	0.2237	1.1270	0.1221	0.4475	0.0783	11,566	1,251	383
Protein modification	3.4352	0.2075	1.8142	0.1660	0.5022	0.0530	2,308	468	383
Transposable elements	1.9966	0.2085	1.8702	0.2045	0.6045	0.0701	1,394	636	182
Unknown conserved	3.0053	0.0835	1.7034	0.0644	0.7092	0.0417	1,035	101	2330
Immunity	2.5761	0.3077	2.8458	0.4407	0.8456	0.1821	697	308	100
Secreted	1.8015	0.0488	2.7768	0.0691	1.3630	0.0453	2,770	140	4419
Unknown	1.2875	0.0859	2.8527	0.1544	1.6300	0.1123	1,058	192	952
