# Supplementary material for: Tissue- and time-dependent transcription in Ixodes ricinus salivary glands and midguts when blood feeding on the vertebrate host
Source: Sci Rep. 2015 Mar 13;5:9103. doi: 10.1038/srep09103 (PMC4357865; doi:10.1038/srep09103)
Supplement: Supplementary Information [file srep09103-s1.pdf]

Supplemental Information

Tissue- and time-dependent transcription in *Ixodes ricinus* salivary glands and midguts when blood feeding on the vertebrate host

Michalis Kotsyfakis[1*], Alexandra Schwarz[1], Jan Erhart[1], José M.C. Ribeiro[2]

[1] Institute of Parasitology, Biology Centre of the Academy of Sciences of Czech Republic, Branisovska 31, 37005 Budweis, Czech Republic.

[2] Section of Vector Biology, Laboratory of Malaria and Vector Research, National Institute of Allergy and Infectious Diseases, 12735 Twinbrook Parkway room 2E32D, Rockville MD 20852, USA.

*To whom correspondence should be addressed. Michail Kotsyfakis; Tel: +420-387775492; Fax: +420-385310388; Email: mich_kotsyfakis@yahoo.com

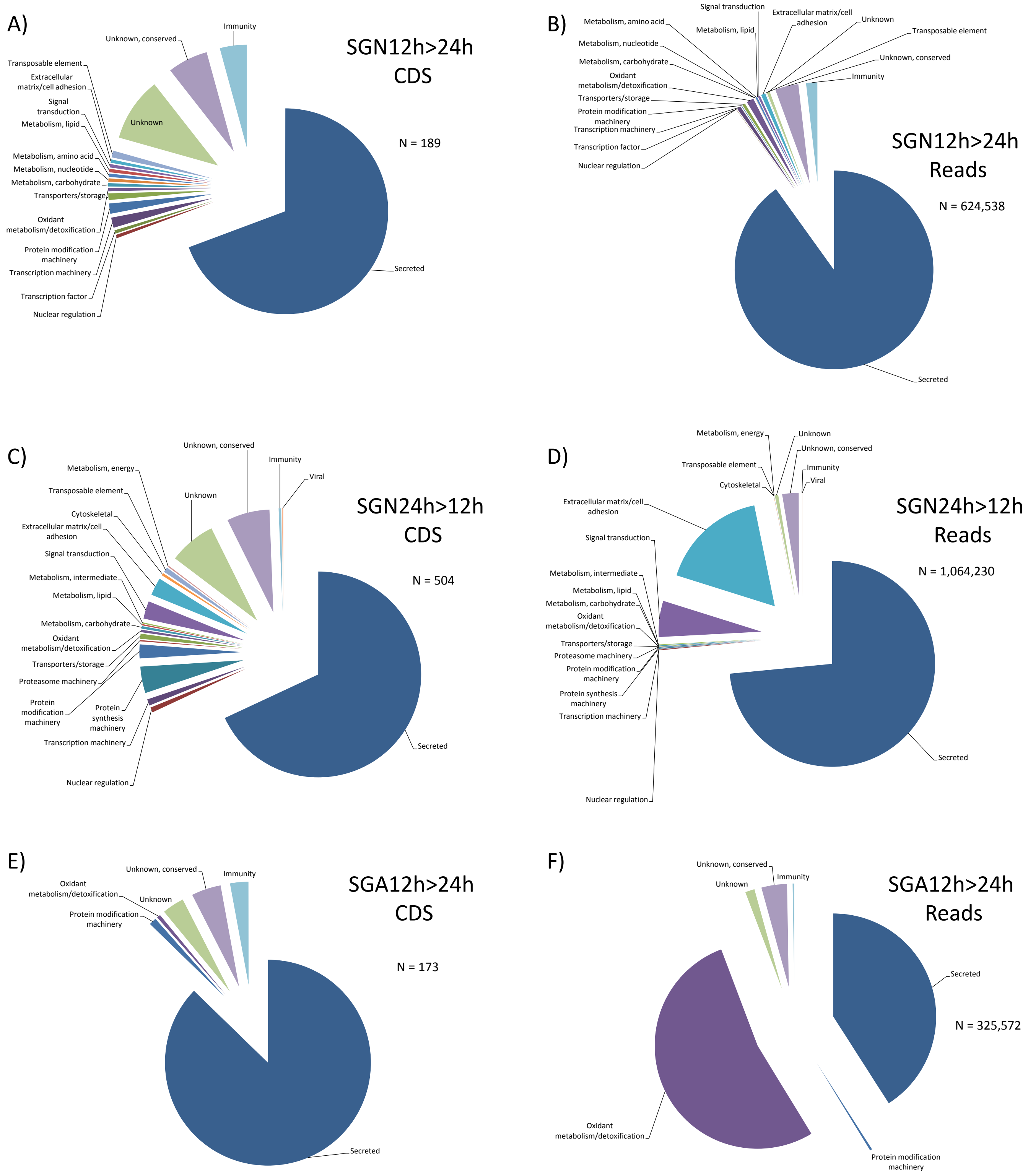

A)
SGN12h>24h
CDS
N = 189
Immunity
Unknown, conserved
Unknown
Transposable element
Extracellular matrix/cell adhesion
Signal transduction
Metabolism, lipid
Metabolism, amino acid
Metabolism, nucleotide
Metabolism, carbohydrate
Transporters/storage
Oxidant metabolism/detoxification
Protein modification machinery
Transcription machinery
Transcription factor
Nuclear regulation
Secreted
B)
SGN12h>24h
Reads
N = 624,538
Signal transduction
Extracellular matrix/cell adhesion
Metabolism, amino acid
Metabolism, lipid
Unknown
Transposable element
Metabolism, nucleotide
Metabolism, carbohydrate
Unknown, conserved
Oxidant metabolism/detoxification
Immunity
Transporters/storage
Protein modification machinery
Transcription machinery
Transcription factor
Nuclear regulation
Secreted
C)
SGN24h>12h
CDS
N = 504
Unknown, conserved
Immunity
Metabolism, energy
Viral
Transposable element
Unknown
Cytoskeletal
Extracellular matrix/cell adhesion
Signal transduction
Metabolism, intermediate
Metabolism, lipid
Metabolism, carbohydrate
Oxidant metabolism/detoxification
Transporters/storage
Proteasome machinery
Protein modification machinery
Protein synthesis machinery
Transcription machinery
Nuclear regulation
Secreted
D)
SGN24h>12h
Reads
N = 1,064,230
Metabolism, energy
Unknown
Unknown, conserved
Transposable element
Immunity
Cytoskeletal
Viral
Extracellular matrix/cell adhesion
Signal transduction
Metabolism, intermediate
Metabolism, lipid
Metabolism, carbohydrate
Oxidant metabolism/detoxification
Transporters/storage
Proteasome machinery
Protein modification machinery
Protein synthesis machinery
Transcription machinery
Nuclear regulation
Secreted
E)
SGA12h>24h
CDS
N = 173
Unknown, conserved
Immunity
Oxidant metabolism/detoxification
Unknown
Protein modification machinery
Secreted
F)
SGA12h>24h
Reads
N = 325,572
Unknown, conserved
Immunity
Unknown
Secreted
Oxidant metabolism/detoxification
Protein modification machinery

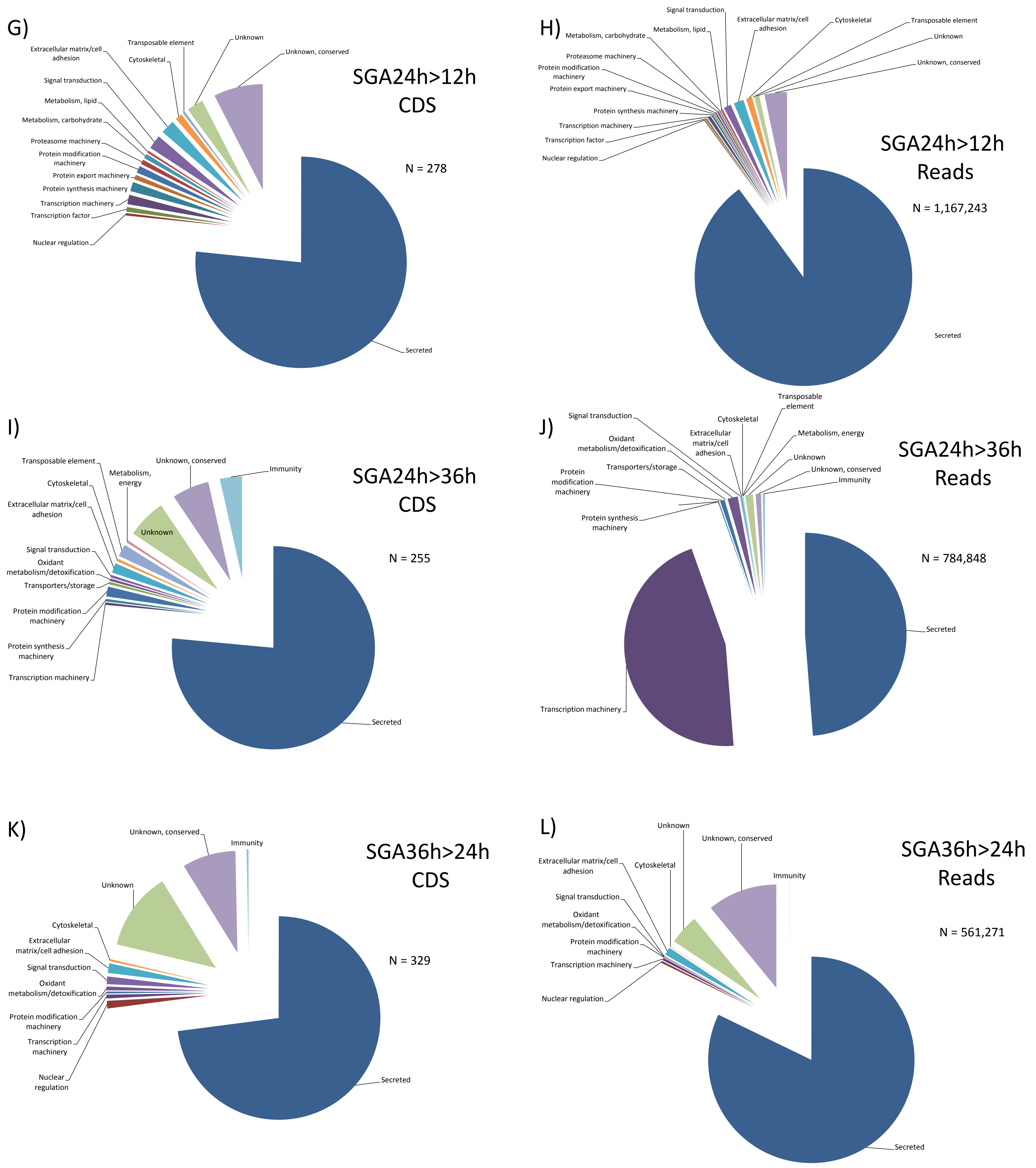

Supplemental Figure S1. All paired salivary gland (SG) time-dependent comparisons for nymphal (N) and adult (A) ticks representing the majority of overexpressed coding sequences (CDS) encoding members of the secreted class. (**A-L**): Pie charts representing the proportion of genes belonging to different functional classes that are ten-fold or more overexpressed in paired comparisons of libraries derived from adult

and nymphal salivary glands as tick feeding progresses over the different experimental time points (12, 24, or 36 hours of feeding). Similar to Figure 1, two different pie charts are constructed for each paired comparison. In (**A**, **C**, **E**, **G**, **I**, **K**), the proportion of the total number of different CDS encoding a polypeptide of the same predicted function is compared to the total number of CDS found in our transcriptomes. In (**B**, **D**, **F**, **H**, **J**, **L**), the proportion of the total number of different sequence reads assigned to CDS encoding a polypeptide of the same predicted function is compared to the total number of mapped sequence reads.

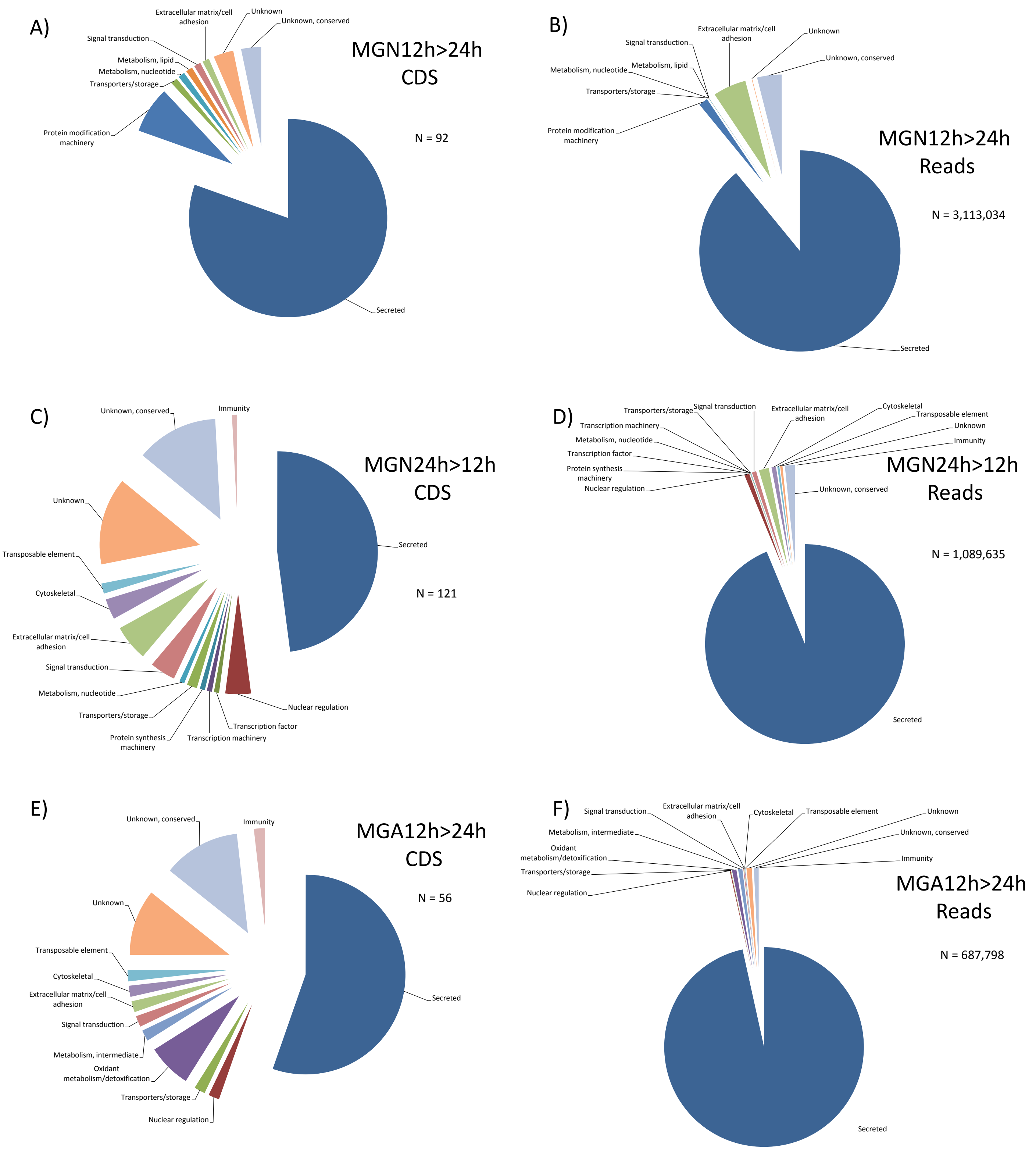
A)
MGN12h>24h
CDS
N = 92
Extracellular matrix/cell adhesion
Unknown
Unknown, conserved
Signal transduction
Metabolism, lipid
Metabolism, nucleotide
Transporters/storage
Protein modification machinery
Secreted
B)
MGN12h>24h
Reads
N = 3,113,034
Extracellular matrix/cell adhesion
Unknown
Signal transduction
Unknown, conserved
Metabolism, lipid
Metabolism, nucleotide
Transporters/storage
Protein modification machinery
Secreted
C)
MGN24h>12h
CDS
N = 121
Unknown, conserved
Immunity
Unknown
Secreted
Transposable element
Cytoskeletal
Extracellular matrix/cell adhesion
Signal transduction
Metabolism, nucleotide
Transporters/storage
Protein synthesis machinery
Transcription machinery
Transcription factor
Nuclear regulation
D)
MGN24h>12h
Reads
N = 1,089,635
Transporters/storage
Signal transduction
Extracellular matrix/cell adhesion
Cytoskeletal
Transposable element
Transcription machinery
Unknown
Metabolism, nucleotide
Immunity
Transcription factor
Protein synthesis machinery
Nuclear regulation
Unknown, conserved
Secreted
E)
MGA12h>24h
CDS
N = 56
Unknown, conserved
Immunity
Unknown
Transposable element
Cytoskeletal
Extracellular matrix/cell adhesion
Signal transduction
Metabolism, intermediate
Oxidant metabolism/detoxification
Transporters/storage
Nuclear regulation
Secreted
F)
MGA12h>24h
Reads
N = 687,798
Signal transduction
Extracellular matrix/cell adhesion
Cytoskeletal
Transposable element
Unknown
Metabolism, intermediate
Unknown, conserved
Oxidant metabolism/detoxification
Immunity
Transporters/storage
Nuclear regulation
Secreted

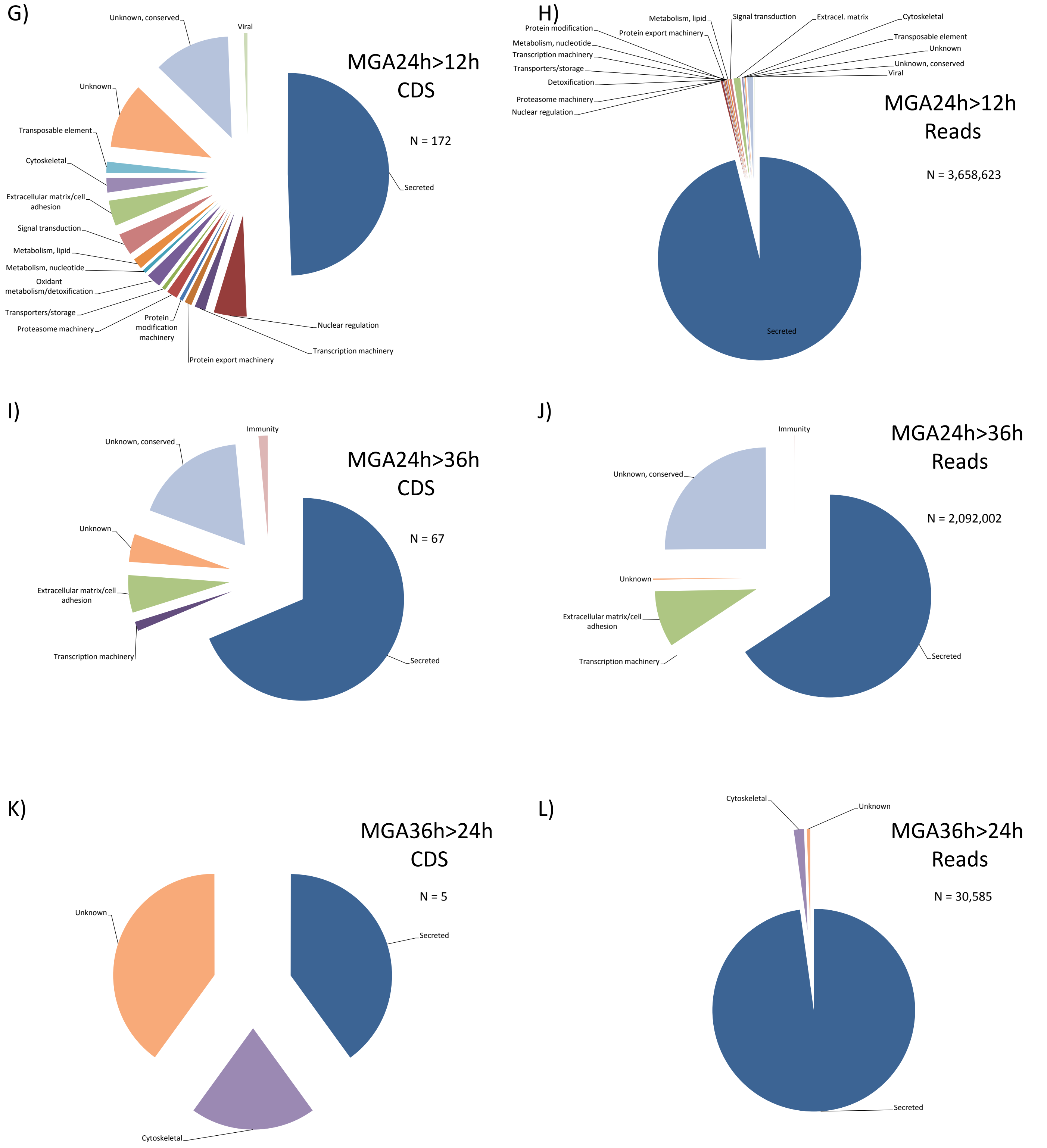

Supplemental Figure S2. All paired midgut (MG) time-dependent comparisons for nymphal (N) and adult (A) ticks representing the majority of overexpressed coding sequences (CDS) encoding members of the secreted class. (**A-L**): Pie charts representing the proportion of genes belonging to different functional

classes that are ten-fold or more overexpressed in paired comparisons of libraries derived from adult and nymphal salivary glands as tick feeding progresses over the different experimental time points (12, 24, or 36 hours of feeding). Similar to Figure 1, two different pie charts are constructed for each paired comparison. In (**A**, **C**, **E**, **G**, **I**, **K**), the proportion of the total number of different CDS encoding a polypeptide of the same predicted function is compared to the total number of CDS found in our transcriptomes. In (**B**, **D**, **F**, **H**, **J**, **L**), the proportion of the total number of different sequence reads assigned to CDS encoding a polypeptide of the same predicted function is compared to the total number of mapped sequence reads.

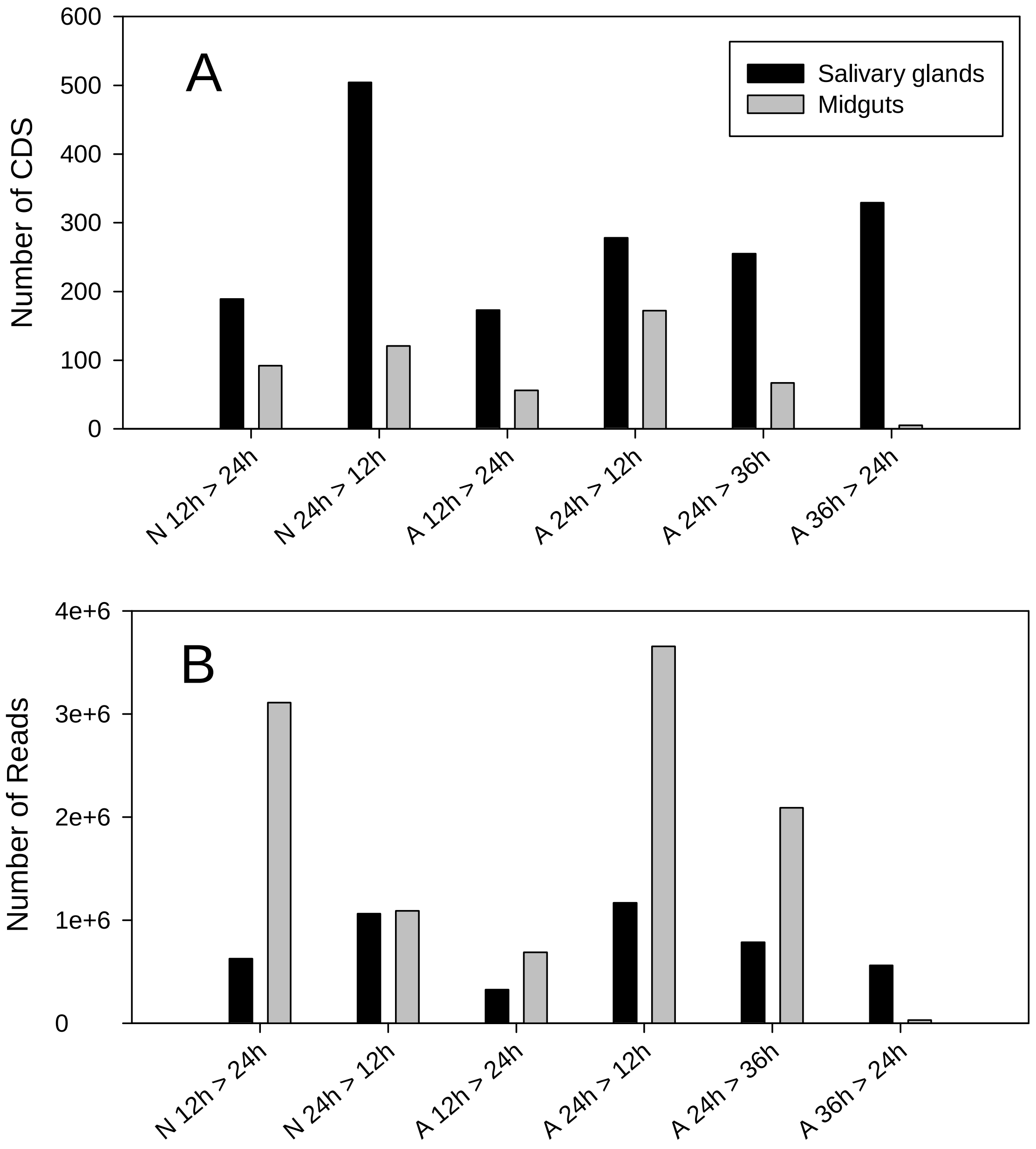

Supplemental Figure S3. Number of *Ixodes ricinus* coding sequences and their associated number of reads that are ten-fold or more overexpressed in paired comparisons of libraries derived from adult and nymphal salivary glands and midguts. (**A**) Overexpressed number of coding sequences (CDS). (**B**) Associated number of reads on overexpressed CDS. N and A indicate tissues from nymphal or adult ticks used for the library comparisons at the indicated times of tissue collection.

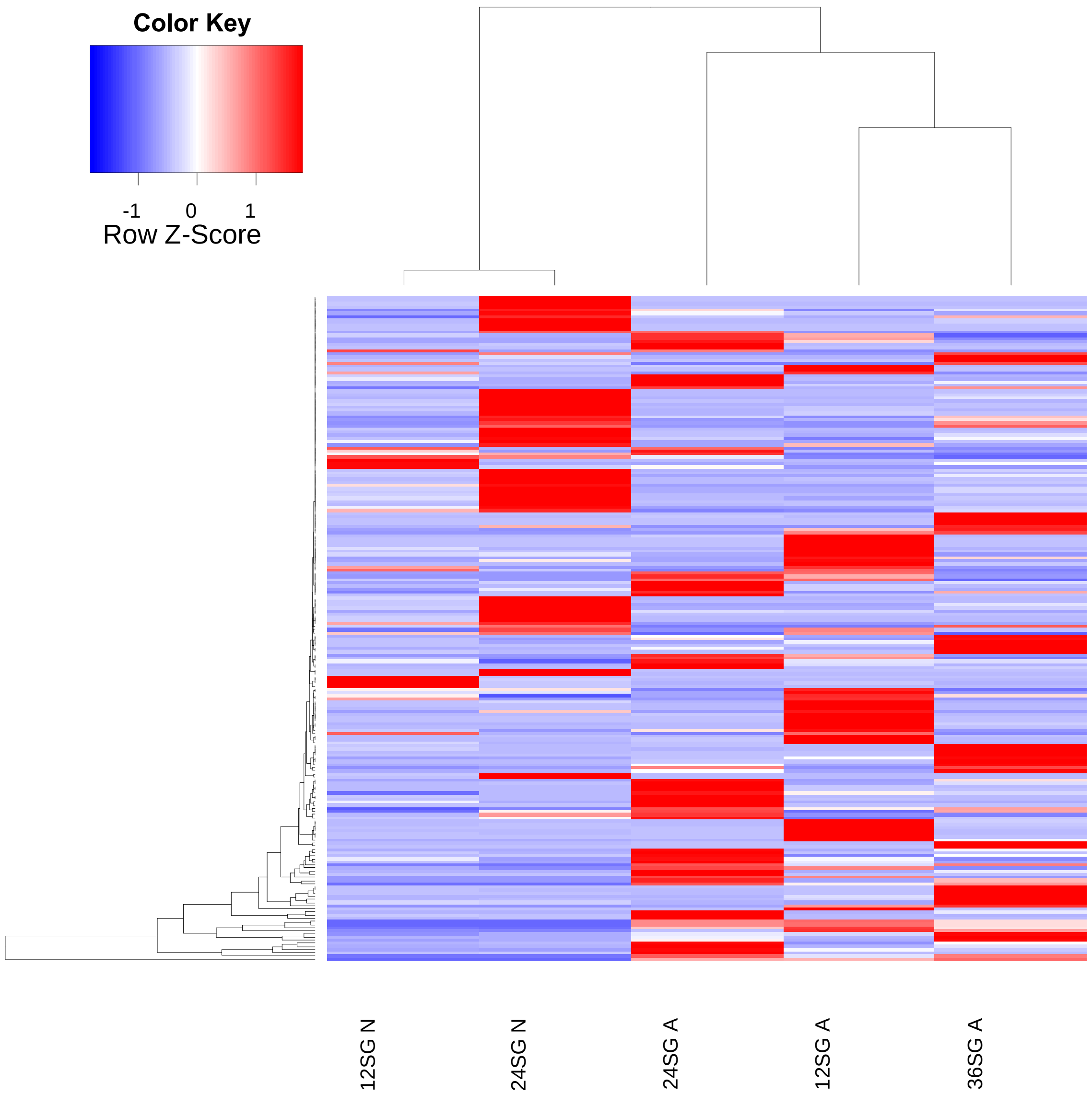

Supplemental Figure S4. Genes encoding salivary Kunitz-domain containing proteins from *Ixodes ricinus* that are at least ten-times differentially expressed at different developmental stages (nymphs or adults) and times post feeding (12, 24, or 36 hours).

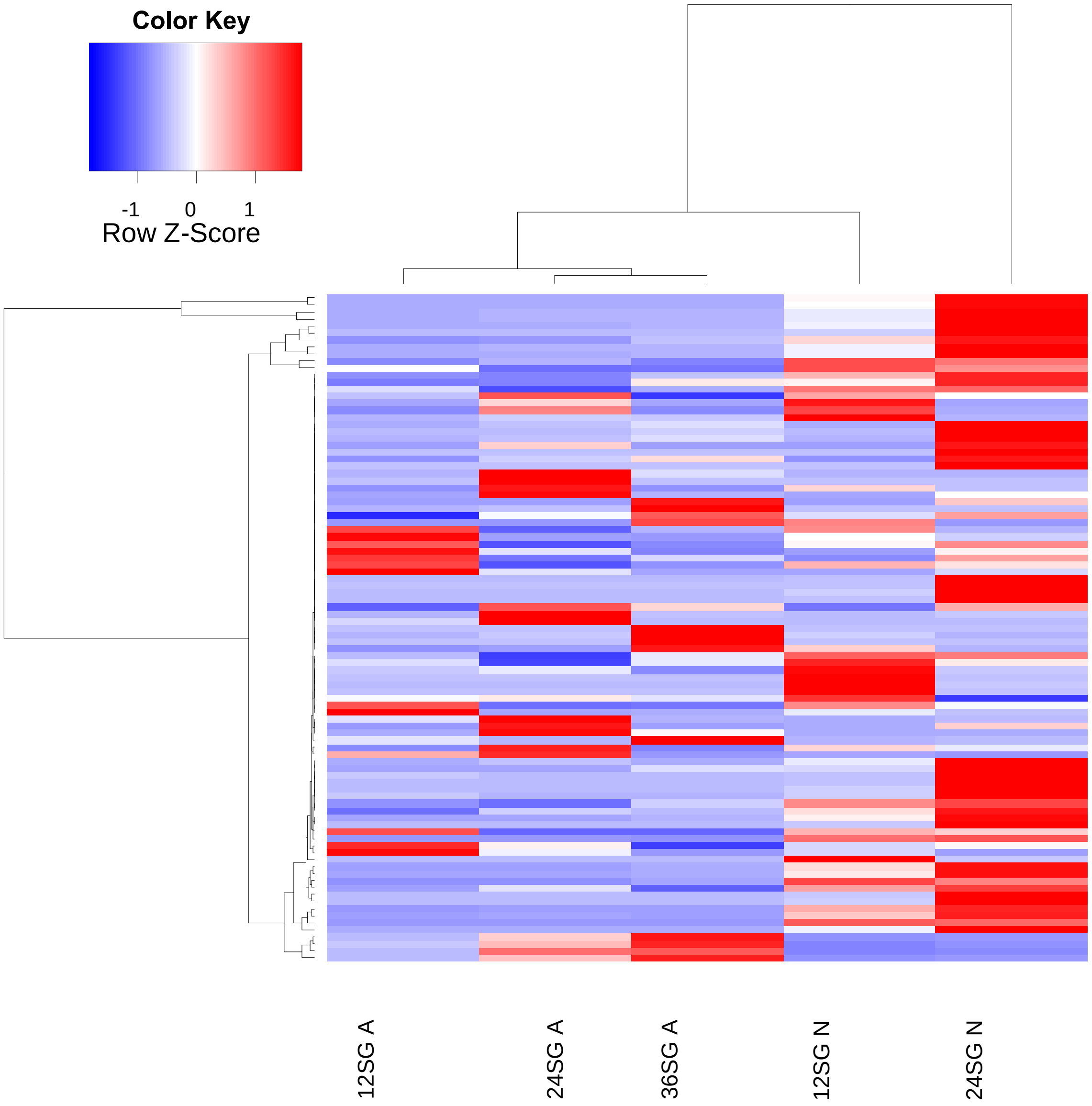

Supplemental Figure S5. Genes encoding salivary lipocalins from *Ixodes ricinus* that are at least ten-times differentially expressed at different tick developmental stages (nymphs or adults) and times post feeding (12, 24, or 36 hours).

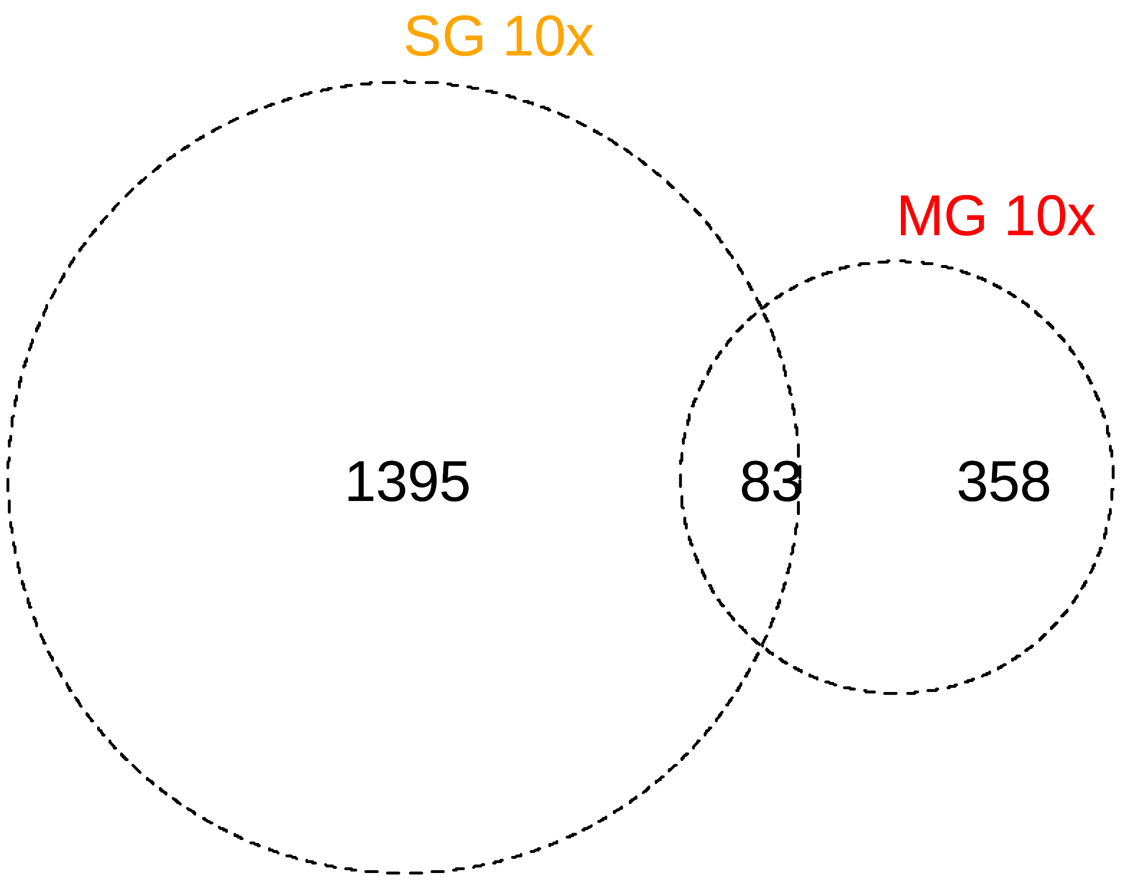

Supplemental Figure S6. Venn diagram indicating overlapping CDS that are at least ten-times differentially expressed in salivary (SG) or midgut (MG) libraries from *Ixodes ricinus*.

Supplemental Tables

Supplemental Table S1. Functional classification of transcripts originating from the assembly of *Ixodes ricinus* salivary and midgut libraries.

Class	Number of contigs	Number of reads	% Total contigs	% Total reads
Secreted	9,048	55,384,419	35.06	37.22
Housekeeping	12,913	88,675,291	50.03	59.59
Transposable element	653	1,193,511	2.53	0.80
Viral	27	29,602	0.10	0.02
Unknown	3,167	3,530,235	12.27	2.37
Total	25,808	148,813,058	100	100

Supplemental Table S2. Functional classification of the coding sequences and their associated number of reads derived from the salivary and midgut transcriptome of *Ixodes ricinus*.

Class	Number of CDS	Number of reads	% Total CDS	% Total reads
**Putative secreted proteins**				
Enzymes				
5' nucleotidase/apyrase	18	5,648	0.0697	0.0038
Endonucleases	11	13,066	0.0426	0.0088
Metalloproteases	330	909,052	1.2787	0.6109
Protease inhibitors				
Salivary TIL domain peptides	53	6,820	0.2054	0.0046
Other TIL domain peptides	11	111,582	0.0426	0.0750
Kunitz-containing peptides	612	4,169,919	2.3714	2.8021
Basic tail	319	10,490,534	1.2361	7.0495
EES tail repeat related to basic tail	49	2,017,703	0.1899	1.3559
Basic tail of argasids and Ixodids	98	85,176	0.3797	0.0572
18.3 kDa	73	1,631,804	0.2829	1.0965
Tick carboxypeptidase inhibitor	2	171	0.0077	0.0001
Serpins	18	33,573	0.0697	0.0226
Cystatins	10	75,609	0.0387	0.0508
Antigen 5 family	37	53,738	0.1434	0.0361
Immunity related				
Ixoderin	58	49,115	0.2247	0.0330
Lysozyme	2	4,436	0.0077	0.0030
Defensins	13	197,850	0.0504	0.1330
Microplusin	22	21,588	0.0852	0.0145
Peptidoglycan recognition protein	34	4,945	0.1317	0.0033
ML domain-containing protein	23	605,643	0.0891	0.4070
5.3 kDa family	97	44,179	0.3759	0.0297
Lipocalins	685	1,375,877	2.6542	0.9246
Isac anticomplement family - Ixodes specific	86	163,576	0.3332	0.1099
Papa family	21	1,398,217	0.0814	0.9396
Ixodegrin/Prokineticin	145	464,169	0.5618	0.3119
Ixodegrins	17	72,274	0.0659	0.0486
Ixostatin/Salp15	110	8,457	0.4262	0.0057
Salp15-2	71	34,627	0.2751	0.0233
Salp-15 3	226	486,051	0.8757	0.3266
Prostriate ixostatins	21	3,746	0.0814	0.0025
Ixostatin/Salp15 superfamily	56	21,152	0.2170	0.0142
Ixostatin 2/Salp15 superfamily	50	59,585	0.1937	0.0400
8.9 kDa family	111	350,931	0.4301	0.2358
8.9 kDa family - 2	33	66,852	0.1279	0.0449
GYY/chitin binding	32	48,454	0.1240	0.0326
23 kDa protein	6	11,226	0.0232	0.0075
DAP-36	10	2,773	0.0387	0.0019
Cytotoxin DAP-36 like	42	51,164	0.1627	0.0344
DAP-36 like - midgut specific	21	702,228	0.0814	0.4719
Ixodes 10 kDa peptide family	264	182,251	1.0229	0.1225
Acid tail Ixodes family	41	20,251	0.1589	0.0136

8 cys family	27	3,736	0.1046	0.0025
WC family	21	157,584	0.0814	0.1059
LTL family	16	43,762	0.0620	0.0294
P32 family	161	27,307	0.6238	0.0183
Fam40-17	54	2,672	0.2092	0.0018
Small GGY peptide < 10 kDa	52	435,370	0.2015	0.2926
Cuticle-like Ala rich family found in Pro/Metastriates	35	152,601	0.1356	0.1025
Other glycine rich proteins	11	161,391	0.0426	0.1085
Ixodes 14 kda family	20	95,181	0.0775	0.0640
Other secreted, highly expressed on SG	64	621,918	0.2480	0.4179
Other secreted proteins overexpressed in SG	865	382,574	3.3517	0.2571
Other secreted proteins overexpressed in midguts	707	13,822,164	2.7395	9.2883
Other secreted proteins	3,307	13,221,579	12.8139	8.8847
**Housekeeping proteins**				
Protein synthesis	534	19,204,663	2.0691	12.9052
Unknown conserved	3,267	12,386,715	12.6589	8.3237
Transcription machinery	922	7,127,441	3.5725	4.7895
Signal transduction	1,465	6,793,519	5.6765	4.5651
Protein modification	573	6,415,897	2.2202	4.3114
Energy metabolism	252	4,463,958	0.9764	2.9997
Extracellular matrix	244	4,022,857	0.9454	2.7033
Cytoskeletal	335	3,512,716	1.2980	2.3605
Transporters and channels	869	3,198,212	3.3672	2.1491
Peritrophins - chitin binding	45	3,058,983	0.1744	2.0556
Lipid metabolism	457	2,752,782	1.7708	1.8498
Protein export	411	2,734,465	1.5925	1.8375
Carbohydrate metabolism	277	2,328,346	1.0733	1.5646
Proteasome machinery	333	2,127,483	1.2903	1.4296
Nuclear regulation	393	1,804,927	1.5228	1.2129
Detoxification	357	1,553,089	1.3833	1.0437
Storage	19	1,375,950	0.0736	0.9246
Nucleotide metabolism	173	1,027,326	0.6703	0.6903
Amino acid metabolism	125	1,020,174	0.4843	0.6855
Transcription factor	253	782,480	0.9803	0.5258
Intermediate metabolism	49	447,194	0.1899	0.3005
Immunity	151	289,413	0.5851	0.1945
Nuclear export	42	245,922	0.1627	0.1653
**Unknown**	3,056	3,510,891	11.8413	2.3593
**Unknown possible artifact from polyA**	1,254	279,115	4.8590	0.1876
**Unknown midgut specific proteins**				
13 kDa family	9	777,572	0.0349	0.5225
**Transposable element**	647	371,964	2.5070	0.2500
**Viral**	18	15,153	0.0697	0.0102
**Total**	25,808	148,813,058	100	100

Supplemental Table S3. Functional classification of salivary gland expressed coding sequences (CDS) ten-fold or more overexpressed compared to the midgut libraries.

Class	Number of CDS
**Secreted**	
5' nucleotidase/apyrase	18
Endonucleases	11
Metalloproteases	281
Antigen 5 family	73
Ixoderin	48
Defensins	1
Lipocalin	566
Salivary TIL domain peptides	21
Cystatins	1
Serpins	1
Kunitz-containing peptides	492
Basic tail	286
EES tail repeat related to basic tail	40
Basic tail of argasids and Ixodids	75
18.3 kDa	69
Tick carboxypeptidase inhibitor	81
Papa family	21
Ixodegrin/Prokineticin	137
Ixodegrins	14
Ixostatin/Salp15	63
Salp15-2	52
Salp-15 3	220
Prostriate ixostatins	19
Ixostatin/Salp15 superfamily	53
Ixostatin 2/Salp15 superfamily	38
8.9 kDa family	92
8.9 kDa family - 2	28
GYY/chitin binding	1
23 kDa protein	6
DAP-36	9
Cytotoxin DAP-36 like	33
Microplusin	3
Ixodes 10 kDa peptide family	201
5.3 kDa family	75
Acid tail Ixodes family	37
8 cys family	18
WC family	21
LTL family	16
P32 family	113
Fam40-17	40
Small GGY peptide < 10 kDa	23
Cuticle-like Ala rich family found in Pro/Metastriates	1
Other glycine rich proteins	3

Ixodes 14 kda family	20
Other secreted, highly expressed on SG	64
Other secreted proteins overexpressed in SG	865
Total	***4,332***
**Housekeeping**	
Cytoskeletal	3
Detoxification	34
Extracellular matrix	29
Immunity	20
Amino acid metabolism	3
Carbohydrate metabolism	15
Energy metabolism	3
Intermediate metabolism	1
Lipid metabolism	52
Nucleotide metabolism	6
Nuclear regulation	7
Protein export	13
Protein modification	133
Proteasome machinery	1
Protein synthesis	30
Signal transduction	29
Transcription factor	6
Transcription machinery	17
Transporters and channels	71
Unknown conserved	174
Unknown	338
Unknown possible artifact from polyA	54
Total	***1,039***
**Transposable element**	13
**Viral**	4
**Total**	**5,406**

Supplemental Table S4. Functional classification of midgut expressed coding sequences (CDS) ten-fold or more overexpressed compared to the salivary libraries.

Class	Number of CDS
**Secreted**	
Metalloproteases	2
Antigen 5 family	9
Defensins	5
Lipocalin	1
Other TIL domain peptides	9
Cystatins	5
Serpins	4
Kunitz-containing peptides	22
Ixodegrin/Prokineticin	1
DAP-36 like - midgut specific	21
Microplusin	5
Small GGY peptide < 10 kDa	2
Other secreted proteins overexpressed in midguts	707
Total	***793***
**Housekeeping**	
Cytoskeletal	73
Detoxification	148
Extracellular matrix	60
Perithrophins - chitin binding	41
Immunity	44
Amino acid metabolism	30
Carbohydrate metabolism	62
Energy metabolism	20
Intermediate metabolism	11
Lipid metabolism	111
Nucleotide metabolism	23
Nuclear export	2
Nuclear regulation	59
Protein export	32
Protein modification	52
Proteasome machinery	36
Protein synthesis	14
Signal transduction	324
Storage	9
Transcription factor	34
Transcription machinery	38
Transporters and channels	137
Unknown conserved	401
Unknown	309
Unknown possible artifact from polyA	5
13 kDa family	9
Total	***1,718***
**Transposable element**	63
**Viral**	3
**Total**	**2,943**

Supplemental Table S5. Functional classification of coding sequences (CDS) derived from the salivary gland transcriptomes of *Ixodes ricinus* that are at least ten-fold overexpressed in paired library comparisons.

Class	Number of CDS
**Secreted**	
Metalloproteases	16
Antigen 5 family	6
Ixoderin	20
Lipocalin	95
Salivary TIL domain peptides	8
Serpins	2
Kunitz-containing peptides	212
Basic tail	40
EES tail repeat related to basic tail	20
Basic tail of argasids and Ixodids	21
18.3 kDa	7
Tick carboxypeptidase inhibitor	11
Papa family	2
Ixodegrin/Prokineticin	34
Ixodegrins	3
Ixostatin/Salp15	20
Salp15-2	18
Salp-15 3	59
Prostriate ixostatins	2
Ixostatin/Salp15 superfamily	21
Ixostatin 2/Salp15 superfamily	12
8.9 kDa family	17
8.9 kDa family - 2	8
GYY/chitin binding	13
DAP-36	1
Cytotoxin DAP-36 like	3
Microplusin	2
Ixodes 10 kDa peptide family	88
5.3 kDa family	33
Acid tail Ixodes family	17
8 cys family	13
WC family	3
P32 family	31
Fam40-17	12
Small GGY peptide < 10 kDa	5
Cuticle-like Ala rich family found in Pro/Metastriates	21
Other glycine rich proteins	5
Ixodes 14 kda family	1
Other secreted, highly expressed on SG	12
Other secreted proteins overexpressed in SG	152
Other secreted proteins overexpressed in midguts	12
Other secreted proteins	57

Total	***1,135***
**Housekeeping**	
Cytoskeletal	7
Detoxification	7
Extracellular matrix	13
Peritrophins - chitin binding	1
Immunity	4
Amino acid metabolism	1
Carbohydrate metabolism	5
Energy metabolism	2
Intermediate metabolism	1
Lipid metabolism	3
Nucleotide metabolism	1
Nuclear regulation	8
Protein export	2
Protein modification	18
Proteasome machinery	3
Protein synthesis	25
Signal transduction	25
Transcription factor	3
Transcription machinery	14
Transporters and channels	14
Unknown conserved	63
Unknown	98
Unknown possible artifact from polyA	13
Total	***331***
**Transposable element**	11
**Total**	**1,477**

Supplemental Table S6. Functional classification of coding sequences (CDS) derived from the midgut transcriptomes of *Ixodes ricinus* that are at least ten-fold overexpressed in paired library comparisons.

Class	Number of CDS
**Secreted**	
Endonucleases	1
Metalloproteases	9
Antigen 5 family	1
Lysozyme	1
Defensins	1
Microplusin	2
Lipocalin	11
Kunitz-containing peptide	38
Basic tail	28
EES tail repeat related to basic tail	2
Basic tail of argasids and Ixodids	4
18.3 kDa	13
Papa family	7
Ixodegrin/Prokineticin	9
Ixodegrins	1
Salp15-2	1
Salp-15 3	17
8.9 kDa family	3
GYY/chitin binding	9
23 kDa protein	1
Cytotoxin DAP-36 like	2
DAP-36 like - midgut specific	2
Ixodes 10 kDa peptide family	4
WC family	1
Small GGY peptide < 10 kDa	7
Cuticle-like Ala rich family found in Pro/Metastriates	13
Other glycine rich proteins	3
Other secreted, highly expressed on SG	3
Other secreted proteins overexpressed in SG	4
Other secreted proteins overexpressed in midguts	36
Other secreted proteins	35
Total	***269***
**Housekeeping**	
Cytoskeletal	6
Detoxification	8
Extracellular matrix	9
Perithrophins - chitin binding	1
Immunity	3
Intermediate metabolism	1
Lipid metabolism	6
Nucleotide metabolism	2
Nuclear regulation	12
Protein export	2
Protein modification	2

Proteasome machinery	3
Protein synthesis	1
Signal transduction	10
Transcription factor	1
Transcription machinery	5
Transporters and channels	7
Unknown conserved	42
Unknown	40
Unknown possible artifact from polyA	2
13 kDa family	1
Total	***164***
**Transposable element**	6
**Viral**	1
**Total**	**440**

Supplemental Table S7. Functional classification of salivary coding sequences (CDS) that were ten-fold or more overexpressed in both the salivary gland and midgut libraries at different time points.

Class	Number of CDS
**Putative secreted proteins**	
Metalloproteases	1
Lipocalin	2
Kunitz-containing peptides	15
Basic tail	2
EES tail repeat related to basic tail	1
Basic tail of argasids and Ixodids	1
18.3 kDa	1
Ixodegrin/Prokineticin	1
Ixodegrins	1
Salp-15 3	3
GYY/chitin binding	7
Cytotoxin DAP-36 like	1
Ixodes 10 kDa peptide family	3
Small GGY peptide < 10 kDa	3
Cuticle-like Ala rich family found in Pro/Metastriates	12
Other glycine rich proteins	3
Other secreted proteins overexpressed in SG	2
Other secreted proteins overexpressed in midguts	4
Other secreted proteins	9
Immunity	2
Total	***74***
**Housekeeping proteins**	
Transcription machinery	1
Unknown conserved	3
Unknown	5
Total	***9***
**Total**	**83**
